# Secondary Hemophagocytic Syndrome in a Patient with Plasma Cell Myeloma and CNS Involvement Treated with Lenalidomide

**DOI:** 10.3390/medicina58101350

**Published:** 2022-09-26

**Authors:** Sławomir Milczarek, Piotr Kulig, Bartłomiej Baumert, Aleksandra Łanocha, Krzysztof Sommerfeld, Ewa Borowiecka, Bogumiła Osękowska, Edyta Paczkowska, Barbara Zdziarska, Bogusław Machaliński

**Affiliations:** 1 Department of General Pathology, Pomeranian Medical University, 70-111 Szczecin, Poland; 2 Department of Hematology and Transplantology, Pomeranian Medical University, 71-252 Szczecin, Poland

**Keywords:** multiple myeloma, hemophagocytic lymphohistiocytosis, bone marrow transplantation, chemotherapy

## Abstract

We present an extremely rare case report of a 29-year-old multiple myeloma patient with central nervous system involvement and secondary hemophagocytic lymphohistiocytosis (HLH). We observed that HLH was presumably triggered by the immunomodulatory drug—lenalidomide. HLH is frequently misdiagnosed or underdiagnosed. As HLH requires immediate treatment, our report emphasizes the need to consider HLH in the differential diagnosis when the condition of a patient receiving chemotherapy rapidly deteriorates and an infectious etiology is excluded. We furthermore discuss the pathogenesis of HLH, with particular emphasis on drugs affecting the immune system as well as possible therapeutic strategies.

## 1. Introduction

Plasma cell myeloma (PCM) is a malignancy which contributes to approximately 10% of hematological neoplasms and 1% of all cancers [1]. It is characterized by infiltration of the bone marrow (BM) by malignant plasma cells (PCs) that typically secrete monoclonal protein, i.e., immunoglobulins, heavy or light chains [2]. Central nervous system involvement in the course of PCM (CNS PCM) is rare and occurs in about 1% of cases. The risk of CNS infiltration is associated with high-risk chromosomal abnormalities, plasmablastic morphology and extramedullary manifestations [3]. Hemophagocytic lymphohistiocytosis (HLH) is a rare, life-threatening condition with persistent hyperinflammation and overactivation of the immune system. The uncontrolled activation of T lymphocytes and macrophages leads to a massive cytokine release, resulting in hemophagocytosis of the hematopoietic cells. HLH can be primary, i.e., arise from a genetic defect, commonly seen in pediatric patients, or secondary to a pre-existing condition that triggers the pathology, usually in the adult population [4]. Common clinical symptoms include fever, hepatosplenomegaly and cytopenia. Laboratory findings include hypertriglyceridemia, coagulopathy with hypofibrinogenemia, elevated serum ferritin and transaminases [5].The diagnosis is confirmed when the causative gene is identified or the patient meets the generally accepted criteria [5]. Therapeutic options include immunosuppression, chemotherapy and allogeneic hematopoietic stem cell transplantation (alloHSCT) [6]. Cases of HLH in patients with PCM are anecdotal, and to the best of our knowledge, our case represents the only report of secondary HLH in a CNS PCM patient with an accumulation of possible triggering mechanisms.

## 2. Case Report

**Figure 1:**
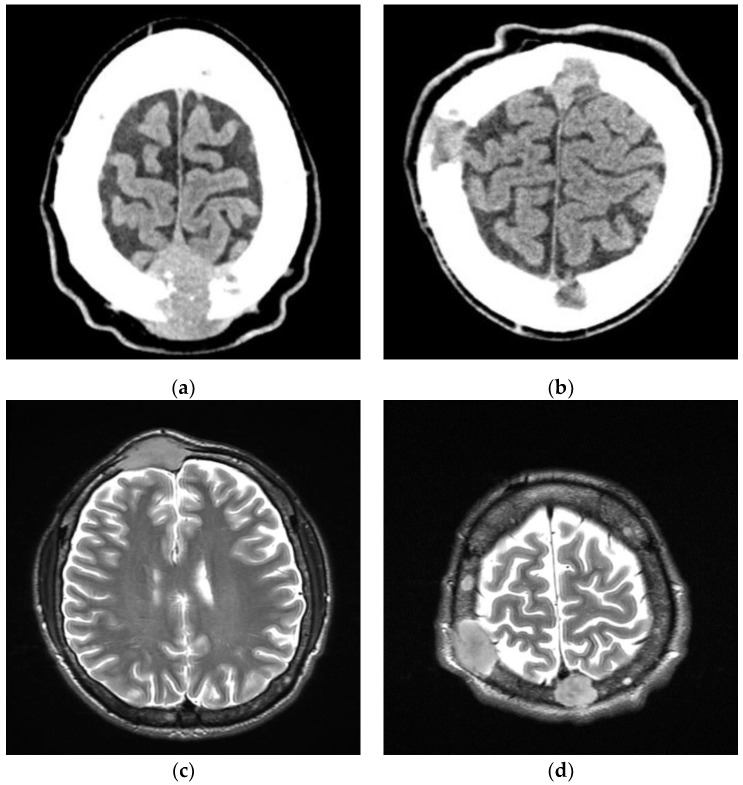
CT (**a**,**b**) and MRI (**c**,**d**) scans of plasma cell tumor infiltrating central nervous system (CNS).

We present a case report of a 29-year-old patient with CNS PCM and secondary HLH. Initially, PCM was manifested by severe back pain, unintentional weight loss (approx. 10 kg) and the appearance of a fronto-parietal tumor (Figure 1). MRI of the axial skeleton revealed a thoracic spine tumor (Th10-12) infiltrating the spinal canal and numerous osteolytic lesions throughout the spine. Initial laboratory tests showed normocytic anemia and high erythrocyte sedimentation rate. Specific tests revealed the presence of monoclonal IgG kappa in the immunofixation assay, high monoclonal protein concentration up to 7.7 g/dL, abnormal kappa/lambda ratio of 0.059 and hyperproteinemia. Histopathology revealed infiltration of malignant plasma cells (CD138+, CD38+, lambda > kappa) exceeding 85% of BM cells. Clonality was confirmed using cytometric evaluation. High-risk cytogenetic abnormalities were excluded. Whole-body low-dose CT scans confirmed countless osteolytic lesions, with particular emphasis on the spine and long bones. MRI of the head revealed tumors with osteodural infiltrations (Figure 1).

**Figure 2:**
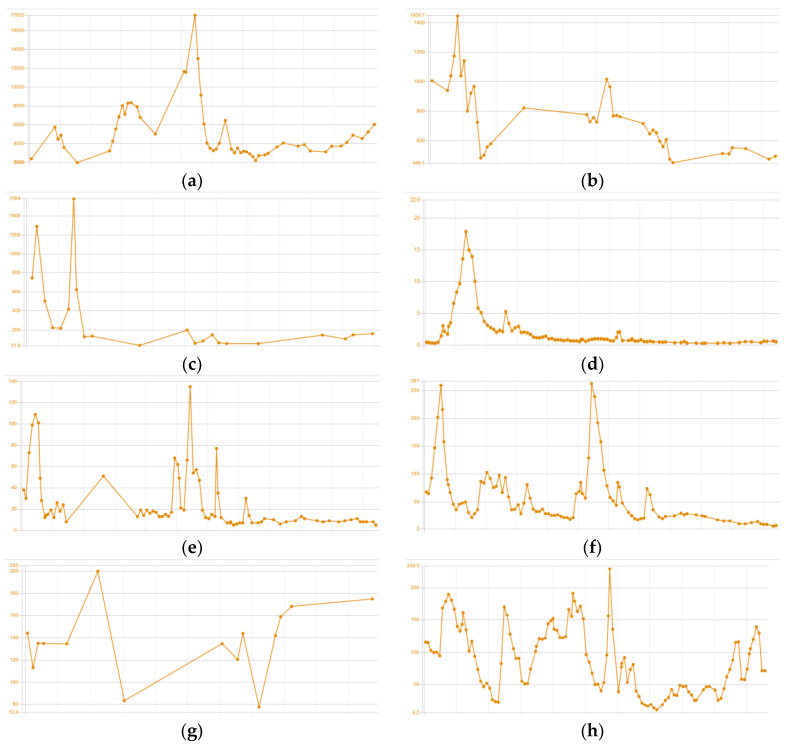
Trend chart of HLH-relatedlaboratory tests: (**a**)ferritin [μ/L]; (**b**) fibrinogen [mg/dL]; (**c**) interleukin 6 [pg/mL]; (**d**) bilirubin [mg/dL]; (**e**) aspartate aminotransferase [IU/L]; (**f**) alanine aminotransferase [IU/L]; (**g**) triglycerides [mg/dL]; (**h**) c-reactive protein [mg/L]. Each box on the *x*-axis corresponds to 1 day.

**Figure 3:**
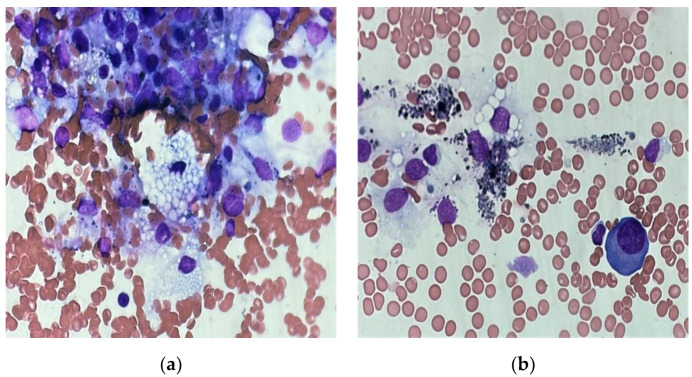

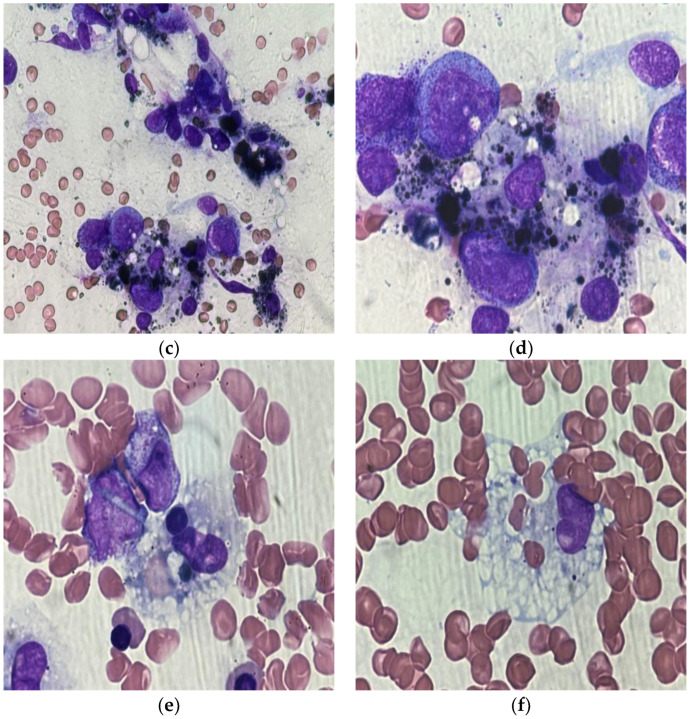
Hemophagocytic lymphohistiocytosis (**a**–**f**) (HLH) in patient with plasma cell myeloma. Bone marrow aspirate smear depicting hemophagocytosis, macrophages engulfing erythrocytes and erythroblasts (different magnitudes).

Due to the high risk of paraplegia, an urgent spondylodesis with tumor reduction was performed. Systemic treatment included a triplet VTD regimen with adjuvant IMRT for the most prominent bone. After six cycles of VTD chemotherapy, the patient achieved biochemical complete remission with partial remission (PR) by MRI. Overall, the depth of the response was classified as PR according to IMWG criteria. The patient was eligible for a stem cell transplant and the auto-allo platform was scheduled. The autologous transplantation (ASCT) was performed with MEL200 used as preparative regimen. Three months after the transplant, the patient relapsed. Clinical symptoms included bone pain, head tumor enlargement, fever up to 39 °C and 5 kg weight loss. PET/CT detected new osteolytic lesions and progression of the existing ones. Specific laboratory tests corresponded to imaging tests showing apparent progression. The patient received treatment according to the DaraVD protocol. During treatment, the patient experienced further clinical deterioration, with progression of PCM and developed progressive cytopenia. Plasma cell leukemia was excluded, as no plasma cells were detected in the peripheral blood smear. The concentration of monoclonal protein increased to 1.9 g/dL. Taking into account the rapid progression, it was decided to initiate the KRD-PACE protocol. However, grade 3 hepatotoxicity was noted after one day of the new chemotherapy regimen. Therefore, K-PACE was withdrawn and the patient continued treatment with lenalidomide only, as it was assumed to be the most neutral. In the following days, high fever, pancytopenia, elevated liver function test, hypophosphatemia, hypofibrinogenemia and hyperferritinemia occurred (Figure 2). There was a reduction in tumor size and monoclonal protein concentration (0.2 g/dL). Secondary HLH was suspected; however, the diagnosis was not yet confirmed. Therefore, treatment was initiated according to the cytokine release syndrome protocol. The subsequent BM biopsy confirmed the diagnosis of HLH (Figure 3). Lenalidomide was withdrawn and HLH treatment was initiated in accordance with HLH-2004 guidelines, resulting in clinical improvement and symptoms resolution. As it was hypothesized that HLH could be triggered by lenalidomide or the active PCM itself, it was decided to rechallenge KD-PACE (without lenalidomide) as a subsequent chemotherapy cycle. Assessment on day 15 revealed hypoplastic BM with an infiltration of atypical PCs and the presence of hemophagocytic cells. Additionally, biochemical progression was noted. KD-PACE was ineffective and the exact etiology of HLH remained uncertain. The previously effective lenalidomide was resumed, along with HLH treatment. We observed a satisfactory response to the treatment with improvement in specific parameters, but at the same time, symptoms of HLH relapsed. After discontinuation of lenalidomide, PCM progressed—the skull tumor enlarged and new periorbital lesions appeared. Due to the PCM progression and probable causal relationship between lenalidomide and HLH, isatuximab/bendamustine/dexamethasone chemotherapy was initiated with concomitant HLH treatment, leading to PR of PCM. Of note, the clinical symptoms of HLH also resolved and an incomplete recovery of hematopoiesis was achieved. The patient remained dependent on platelet transfusions. In the aftermath of an overall satisfactory response, he proceeded to matched-sibling allo-HSCT. Conditioning consisted of bendamustine and total body irradiation. The procedure was complicated by primary graft failure and a lack of thrombopoiesis, and the patient remained dependent on platelet transfusions at discharge. Four weeks after discharge from the hospital, the disease relapsed. Due to the exhaustion of all therapeutic options, the patient was referred to palliative care and died eight weeks later.

## 3. Discussion

HLH is a life-threatening disorder, often misdiagnosed, which contributes to its high mortality. There are many putative etiologies of secondary HLH. The most common are infections, malignancies, autoimmune disorders and immunosuppression. In the adult population, hematological malignancies are the most common predisposing factors, while drugs and viral infections most often trigger the onset of the disease [7]. It is commonly believed that the role of hyperactivated T cells and macrophages is of paramount importance in the development of HLH [8], and the abovementioned immune system dysregulation may be triggered by a wide variety of causes. Patients with neoplasms, especially lymphomas, are most prone to HLH. HLH is estimated to affect 1% of adults with hematological cancers, but the prevalence rises to 20% in patients with certain types of B-cell and T-cell lymphomas. Interestingly, PCM contributes to <1% of the causes of HLH in patients with lymphoproliferative disorders [7] Malignancy-associated HLH (Mal-HLH) remains the most common form of secondary HLH and has the worst prognosis of all HLH subgroups [7]. The PubMed database contains scarce data on HLH in PCM patients. All published cases are very heterogeneous and differ in terms of PCM staging during diagnosis andtreatment protocol, as well as PCM staging at HLH diagnosis. There is limited data that PCM drugs interfere with the immune system and therefore can also trigger HLH. Busch and colleagues conducted a study that investigated the effect of lenalidomide on the immune system. The study concluded that lenalidomide induced both activating and inhibiting components of the immune system, indicating the existence of potential counter-regulatory mechanisms [9]. In addition, another study concluded that lenalidomide treatment was associated with the increase in HLA-DR+ T cells, regulatory T cells and NK cells [10,11]. In this patient, the major pathophysiologic mechanism, thought to be responsible for triggering secondary HLH, was the immunomodulatory drug lenalidomide, which could alter T cell function and aggravate immune dysregulation. However, data supporting a causal relationship between lenalidomide and HLH are limited [12]. The fact that HLH resolved after definite withdrawal of lenalidomide further supports this assumption. Additionally, we recognize that there may have been several mechanisms that contributed to the patient’s altered immunity. One of the postulated HLH triggers is the inability of NK cells to induce lysis of activated T cells and histiocytes. Both lenalidomide and daratumumab treatment disrupt immune homeostasis by altering and dysregulating NK cells’ function, and therefore may contribute to the onset of the disease [13].

## 4. Conclusions

The diagnosis of HLH requires immediate treatment. Mal-HLH should always be suspected when infectious causes are excluded. Although the heterogeneity of the disease renders it difficult to formulate clear recommendations, it is generally accepted that treatment should be balanced between HLH and malignancy-directed therapy. It is recommended to augment cancer-specific treatment with etoposide and cyclosporine. The role of ASCT is unclear, as it may further disrupt immune homeostasis and lower the HLH threshold [14]. Allogeneic transplantation remains the only potentially curative option, offered to carefully selected patients. Although it is not a prevalent condition in PCM patients, it should be always considered when patients exhibit a typical clinical and laboratory profile, especially during PCM treatment that may interfere with the immune system.

## Data Availability

Not applicable.
